# Prevalence of Vancomycin-Resistant Enterococci and Antimicrobial Residues in Wastewater and Surface Water

**DOI:** 10.3390/life11121403

**Published:** 2021-12-15

**Authors:** Kristýna Hricová, Magdaléna Röderová, Petr Fryčák, Volodymyr Pauk, Ondřej Kurka, Kristýna Mezerová, Taťána Štosová, Jan Bardoň, David Milde, Pavla Kučová, Milan Kolář

**Affiliations:** 1 Department of Microbiology, Faculty of Medicine and Dentistry, Palacký University Olomouc, Hněvotínská 3, 77900 Olomouc, Czech Republic; magdalena.roderova@upol.cz (M.R.); kristyna.mezerova@upol.cz (K.M.); tatana.stosova@fnol.cz (T.Š.); jbardon@svuol.cz (J.B.); pavla.kucova@fnol.cz (P.K.); milan.kolar@fnol.cz (M.K.); 2 Department of Analytical Chemistry, Faculty of Science, Palacký University Olomouc, 17. Listopadu 1192/12, 77900 Olomouc, Czech Republic; petr.frycak@upol.cz (P.F.); volodymyr.pauk@upol.cz (V.P.); ondrej.kurka@upol.cz (O.K.); david.milde@upol.cz (D.M.)

**Keywords:** antimicrobial residues, vancomycin-resistant enterococci (VRE), wastewater

## Abstract

Due to the extensive use of antimicrobial agents in human and veterinary medicine, residues of various antimicrobials get into wastewater and, subsequently, surface water. On the one hand, a combination of processes in wastewater treatment plants aims to eliminate chemical and biological pollutants; on the other hand, this environment may create conditions suitable for the horizontal transfer of resistance genes and potential selection of antibiotic-resistant bacteria. Wastewater and surface water samples (Morava River) were analyzed to determine the concentrations of 10 antibiotics and identify those exceeding so-called predicted no-effect environmental concentrations (PNECs). This study revealed that residues of five of the tested antimicrobials, namely ampicillin, clindamycin, tetracycline, tigecycline and vancomycin, in wastewater samples exceeded the PNEC. Vancomycin concentrations were analyzed with respect to the detected strains of vancomycin-resistant enterococci (VRE), in which the presence of resistance genes, virulence factors and potential relationship were analyzed. VRE were detected in 16 wastewater samples (11%) and two surface water samples (6%). The PNEC of vancomycin was exceed in 16% of the samples. Since the detected VRE did not correlate with the vancomycin concentrations, no direct relationship was confirmed between the residues of this antimicrobials and the presence of the resistant strains.

## 1. Introduction

Human health, animal health and a healthy ecosystem are inextricably linked. The international One Health Initiative seeks to promote, improve and protect human and animal health, including the environment they live in. The initiative strives for closer collaboration between human health practitioners, veterinarians and environmental experts to achieve the above goals. An important part of this concept is the issue of infectious diseases, gradually extended to include resistance to antibiotics needed to treat infections [1].

Enterococci are bacteria highly resistant to environmental influences, which allows them to survive in the external environment [2,3]. They are a part of the normal intestinal microflora of both humans and animals but, at the same time, also important bacterial pathogens, especially in humans. The infections caused by them may be both exogenous and endogenous, often nosocomial in nature. The vast majority of human enterococcal infections are caused by the species *Enterococcus faecalis* and *Enterococcus faecium*, with especially the latter being treated with glycopeptides, vancomycin and teicoplanin. Unfortunately, vancomycin-resistant enterococci (VRE) are increasingly common and pose a serious therapeutic problem [4,5,6,7]. There may be numerous sources of VRE, including the human gastrointestinal tract, animals, the hospital environment and wastewater [8,9,10,11,12,13]. The prevalence of VRE in the environment may be related, among other things, to the presence of antimicrobial residues. Antibiotic pollution in the environment (drinking water, surface water, groundwater, wastewater, sludge, soil, etc.) is mainly due to their extensive use in human and veterinary medicine, as well as in aquaculture or agriculture, for the purpose of preventing or treating infections [14]. Antimicrobial agents reach the environment through urine and excreta or through the improper disposal of waste, for example from households. Moreover, various chemicals, including antibiotics, could get into the environment as part of pharmaceutical industry discharges [15].

Wastewater, contaminated by antibiotics and their byproducts, is treated in wastewater treatment plants (WWTPs) by a combination of different processes to eliminate the physical, chemical or biological pollutants and reduce the impact of the effluent on the environment and human health. However, the presence of antibiotics in both metabolized and unmetabolized forms, large amounts of diverse bacterial populations (commensal, pathogenic and environmental) and suitable growth conditions (temperature, pH, high nutrition concentration, etc.) make WWTPs, or other water sources (rivers, lakes, ponds, etc.), an ideal environment for the development of antibiotic-resistant bacteria (ARB). Water ecosystems, in particular WWTPs, are therefore considered one of the main hotspots of potential evolution and spread of ARB and antibiotic resistance genes (ARGs) into the natural environment [13,16,17]. Many studies monitoring the presence of antibiotics in WWTPs have been conducted worldwide [18,19], whereas other studies have paid attention to the occurrence of ARB or ARGs in various water sources (wastewater, drinking water and rivers) [12,13,18,20,21]. According to Bengtsson-Palme et al., antibiotic selection may play a major role in the dissemination of ARB in the environment. Subinhibitory concentrations of antibiotics could have an effect on mutation and recombination rates of bacteria, induce horizontal transfer of genetic material or effect biofilm formation, potentially contributing to persistence of bacteria in biofilms during antibiotic selection [22]. Based on these assumptions, the authors proposed so-called predicted no-effect environmental concentrations (PNECs) for several antibiotics, below which the aforementioned adverse effect of antimicrobial agents will most likely not occur during the exposure in the aquatic environment [23]. Comparing measured levels of individual antibiotics with these values and, at the same time, with the prevalence of ARB may be a valuable tool in predicting the potential selection of resistant bacteria and their impact on the aquatic environment.

As mentioned above, WWTPs are considered to be significant sources and reservoirs of antimicrobial residues (in metabolized or unmetabolized forms), ARB and ARGs which are permanently released into the environment, contaminating surface water, groundwater or agricultural soil. Therefore, it is important to study the association between antimicrobial residues and metabolites in WWTPs and the prevalence of ARB and ARGs.

The study aimed to analyze the presence of selected antibiotics in wastewater and surface water samples, to identify antibiotics exceeding PNECs set by Bengtsson-Palme & Larsson [23] and to investigate the association between the prevalence of VRE and residues of vancomycin, which may be involved in selection of VRE.

## 2. Materials and Methods

### 2.1. Sample Collection

Samples of wastewater were collected at eight sampling sites (Table 1) in the Olomouc region, Czech Republic (250,000 population) between October 2018 and July 2020. The sites were: three sewer drains in the Military Hospital Olomouc, the University Hospital Olomouc (UHO) WWTP (influent + effluent), the Olomouc Municipal WWTP (influent + effluent) and the State Veterinary Institute in Olomouc WWTP effluent. Additionally, surface water samples were collected from the Morava River flowing through the city of Olomouc (before the influent and past the effluent of the Municipal WWTP). Information about the technological principles of WWTPs and positions of the sampling sites are mentioned in the Supplementary Information (Text S1 and Figure S1).

For the detection of residues of selected antimicrobials, approximately one liter of water (wastewater or surface water) from each sampling site was collected into a chemically clean sample bottle, filtered through a membrane filter and transported refrigerated to the laboratory for chemical analysis.

Microbiological analysis aimed to detect VRE started with immersing cellulose swabs (length 11 cm, diameter 5 cm) into wastewater or surface water at all sampling sites, as described by Moore et al. [24]. After 24-h immersion, the swabs were inserted into nutrient broth with 6.5% sodium chloride for 24 h and subsequently inoculated on specific Brilliance VRE Agar chromogenic plates (Oxoid, Brno, Czech Republic) and cultured at 37 °C for 24 h.

### 2.2. Chemical Analysis of Water Samples

#### 2.2.1. Chemicals

The chemicals used were deionized water (from Direct-Q3 UV system, Merck Millipore, Burlington, MA, USA); methanol (for LC-MS, VWR, Střibrná Skalice, Czech Republic); EDTA disodium salt (G.R., Lach-Ner, Neratovice, Czech Republic); hydrochloric acid (p.a., Penta, Chrudim, Czech Republic) and standards of ampicillin, chloramphenicol, clindamycin hydrochloride, erythromycin, linezolid, tetracycline, vancomycin hydrochloride (Spinchem, Plzen, Czech Republic), nitrofurantoin, teicoplanin A2 (mixture of A2-1–A2-5), tigecycline, ampicillin-d5, clindamycin-d3, chloramphenicol-d5, erythromycin-13C,d3, linezolid-d3, nitrofurantoin-13C3, tetracycline-d6 and tigecycline-d9 (LGC standards, Lomianki, Poland).

#### 2.2.2. Preparation of the Internal Standard Solution

Stock solutions of isotopically labeled ampicillin, tetracycline, tigecycline, clindamycin, erythromycin, chloramphenicol, nitrofurantoin and linezolid (each 100 μg/mL in water/methanol 1:1, *v/v*, stored in glass silanized vials) were subjected to an additional intermediate dilution with water/methanol 1:1 (*v/v*) to obtain 1-μg/mL solutions (for all compounds except chloramphenicol, nitrofurantoin and tigecycline). These intermediate solutions (and stock solutions for chloramphenicol, nitrofurantoin and tigecycline) were added to the water/methanol 1:1 (*v/v*) mixture in a polypropylene flask to obtain 10 mL of internal standard solution with the following concentrations: 50-ng/mL ampicillin, 25-ng/mL penicillin G, 50-ng/mL penicillin V, 2.5-ng/mL tetracycline, 1-μg/mL tigecycline, 0.5-ng/mL clindamycin, 7.5-ng/mL erythromycin, 1-μg/mL chloramphenicol, 5-μg/mL nitrofurantoin and 7.5-ng/mL linezolid.

#### 2.2.3. Solid-Phase Extraction (SPE) of Antibiotics from Water Samples

Oasis HLB (Waters, Torrance, CA, USA) SPE cartridges (6 cm^3^, 200 mg) were rinsed with 5 mL of methanol and conditioned with 5 mL of 1% Na_2_EDTA in water acidified with HCl (pH = 3). Each water sample was filtered using a 0.22-μm polypropylene filter into a polypropylene cup. Five milliliters of the filtrate (or an appropriately reduced volume in the case of a reanalysis of samples exceeding the calibration range) was spiked with 100 μL of the internal standard mixture (see above); 556 μL of 10% Na_2_EDTA in water was added to it, and the mixture was acidified with HCl to approx. pH = 3. The resulting solution was loaded onto the SPE column and rinsed with 10 mL of 1% Na_2_EDTA in water acidified with HCl (pH = 3). Then, elution into Protein LoBind tubes (Eppendorf, Hamburg, Germany) was performed using 2 mL of methanol. These extracts were evaporated under nitrogen flow and kept in the freezer (−18 °C) until analysis. On the day of analysis, the extracts were reconstituted in 0.5 mL of a water/methanol/formic acid mixture (84:15:1, *v/v/v*) using a vortex mixer (3000 rpm, 10 s) followed by an ultrasonic bath (5 min). Next, they were centrifuged (13,600× *g*, 5 min), and the supernatants were transferred into 300-µL silanized HPLC vial inserts (Waters, Manchester, UK).

#### 2.2.4. Measurement of Antibiotic Concentrations in Wastewater Samples

Analysis of the antibiotics was performed using the ACQUITY UPLC I-Class chromatographic system coupled to the Xevo TQ-S mass spectrometer with an electrospray ionization source (all Waters, Manchester, UK). Separation was carried out on the Kinetex XB-C18 1.7-µm 100 × 2.1-mm column (Phenomenex, Prague, Czech Republic) kept at 30 °C. A mobile phase consisted of water (A) and methanol (B), both acidified with 0.5% formic acid. The flow rate was set at 0.25 mL/min. The gradient elution started at 5% B, reached 100% B in 3.5 min and was followed by 1.5-min hold and 1.5-min reequilibration. A mixture of water/methanol/formic acid (49:50:1) was used as sample manager purge and wash solvent. An autosampler was equipped with a 50-µL sample loop, and the injection volume was 25 µL. Samples were kept at 10 °C. The column eluate was diverted to waste before 1.3 min and after 5 min to reduce the contamination of the ion source. The mass spectrometric detection was performed in the selected reaction monitoring mode (SRM). Specific SRM transitions with deliberately decreased sensitivity were used for the quantitation of high concentrations. The list of SRM transitions and retention times is provided in Table S2, Supplementary Information. The ionization source conditions for both positive and negative modes were set as follows: ESI voltage 2.0 kV, cone voltage 20 V, source temperature 150 °C, desolvation gas temperature 450 °C, desolvation gas flow 900 L/h, cone gas 150 L/h and nebulizer pressure 5 bar. Nitrofurantoin, chloramphenicol and teicoplanin were ionized in a negative mode; the others were detected in a positive mode.

A set of calibration standards (11 concentration levels) was measured at the beginning of an analytical batch. All real samples were analyzed by two replicate injections per each polarity. A blank sample was injected after each real sample to monitor possible contamination or carryover. QC samples at low, medium or high concentration levels were measured after a sequence of two or three real samples. The data were processed in the TargetLynx v. 4.1 application manager (Waters, Manchester, UK). Mean smoothing with apex peak detection was used for chromatographic peak integration. The retention time window was set to ±0.05 min. The target ion ratio tolerance was set to ±30%. Calibration curves were created using a linear internal calibration method with 1/x^2^ weighting. The calibration curves were considered linear for R^2^ > 0.99. Due to the unreasonably high price of labeled teicoplanin and vancomycin, nitrofurantoin-13C3 and clindamycin-d3 were used as their internal standards (IS), respectively. Erythromycin and its internal standard were detected as the anhydro form due to the rapid degradation in an acidic environment. The limits of detection and quantitation are reported in Table S1, and an example of LC/MS analysis is shown in Figures S2 and S3, Supplementary Information.

### 2.3. Isolation and Identification of VRE

The species identification of suspected isolates grown on Brilliance VRE Agar plates (Oxoid, Brno, Czech Republic) was confirmed using a matrix-assisted laser desorption/ionization time-of-flight mass spectrometer (MALDI-TOF MS) (Biotyper Microflex, Bruker Daltonik GmbH, Bremen, Germany). The obtained isolates were frozen in cryotubes (ITEST plus, Hradec Králové, Czech Republic) and stored at −18 °C until analyzed.

### 2.4. Detection of Antimicrobial Susceptibility

In all isolated enterococci, susceptibility to selected antibiotics (ampicillin, tigecycline, chloramphenicol, tetracycline, clindamycin, vancomycin, teicoplanin, nitrofurantoin, erythromycin and linezolid) was assessed by a standard microdilution method according to the European Committee on Antimicrobial Susceptibility Testing criteria [25]. Quality control was performed using the reference strain *Enterococcus faecalis* ATCC 29212 (CCM, Czech collection of Microorganism, Brno, Czech Republic).

### 2.5. PCR Detection of Resistance Genes and Virulence Factors

Total genomic DNA from VRE isolates was prepared from an overnight culture (16 h, 37 °C) grown on meat–peptone agar using the DNeasy Blood & Tissue Kit (QIAGEN, Hilden, Germany) according to the manufacturer’s recommendations.

Antibiotic resistance genes responsible for resistance to vancomycin (vanA, vanB, vanC1 and vanC2/3); tetracycline (tet(K), tet(L), tet(M), tet(O), tet(S) and tet(X)); macrolides (erm(A), erm(B) and mef(A/E)) and aminoglycosides (ant(4′)-Ia, aac(6′)-aph(2′′) and aph(3′)-IIIa)) were tested by PCR [26,27,28,29,30]. The presence of several tentative virulence genes—gelE (responsible for the production of gelatinase), cylA (cytolysin/hemolysin), asa1 (aggregation substance), hyl (hyaluronidase) and esp (enterococcal surface protein)—was analyzed by multiplex PCR [31].

Polymorphism of the C-terminal region of the *pbp5* gene was investigated according to a study by Poeta et al. [32]. The C-terminal region was amplified by PCR in all VRE isolates using our own designed primer sets targeting the specific area (Table 2).

The reaction mixture and conditions are described in Text S2 (Supplementary Information). Sequencing of single samples was carried out by Elisabeth Pharmacon (Brno, Czech Republic) using amplification primers PBP5 6F and PBP5 11R. Sequence alignment and analysis were performed using the BioEdit sequence alignment editor, version 7.2.5 (Hall, T.A. 1999. BioEdit: a user-friendly biological sequence alignment editor and analysis program for Windows 95/98/NT. Nucl. Acids. Symp. Ser. 41:95-98). The obtained sequence of each isolate was compared with that of the *pbp5* gene sequence included in GenBank (accession no. X84860). The following positive controls were used: *Enterococcus faecalis* NCTC 12201 (NCTC, National Collection of Type Cultures, England) (vanA) and well-characterized strains of *Enterococcus faecalis* (vanB) and *Enterococcus casseliflavus* (vanC1); *Enterococcus faecium* (hyl and esp) and *Enterococcus faecalis* (asa-1, cyl and gelE); *Enterococcus faecium* (tetL, tetM and ermB) and *Enterococcus faecium* (aac(6′)-aph(2′′) and aph(3′)-IIIa) from the collection of the Department of Microbiology, Faculty of Medicine and Dentistry, Palacký University Olomouc. Quality control was performed using a negative control (i.e., water containing no bacterial DNA).

### 2.6. Pulsed-Field Gel Electrophoresis

The similarity of the VRE isolates was performed with pulsed-field gel electrophoresis (PFGE). Bacterial DNA was isolated using a technique described by Husickova et al. and digested by the SmaI restriction endonuclease (New England Biolabs, Ipswich, MA, USA) for 24 h at 25 °C [33]. The obtained DNA fragments were separated by PFGE on 1.2% agarose gel for 24 h at 6 V/cm and pulse times of 2–35 s. Subsequently, the gel was stained with ethidium bromide. The resulting restriction profiles were analyzed with GelCompar II software (Applied Maths, Kortrijk, Belgium). The coefficient of similarity (CS) was calculated using the Dice algorithm. Individual clusters were analyzed with the unweighted pair group method with arithmetic mean algorithm, and the results were interpreted using the criteria defined by Tenover et al. [34]. Optimization and band matching tolerance was set at 1.2%.

## 3. Results and Discussion

The emergence and development of antimicrobial resistance (AMR) in water sources has been the center of attention for numerous research groups in recent years. The authors mainly point to the fact that water sources such as rivers, lakes, ponds and, especially, WWTPs may serve as hotspots for promoting the spread of AMR [13,16,17,35]. At the same time, they may become reservoirs of ARB and ARGs, potentially leading to considerable negative impacts on human and animal health [13,16]. Subinhibitory concentrations of antibiotics could affect mutation and recombination processes in a bacterial population and contribute to induction of the horizontal transfer of genetic material or promote biofilm formation [23].

Over the study period (October 2018 to July 2020), a total of 144 wastewater and 36 river water samples were collected. All were subjected to chemical analysis aimed to detect the presence of residues of selected antimicrobials and to microbiological analysis with subsequent genetic characterization of the obtained VRE.

### 3.1. Detecting the Presence of Selected Antibiotics in Water Samples

The analytical method permitted to quantify the analytes from concentration levels of approximately 1 ng/L (for clindamycin, erythromycin, tetracycline and linezolid); 10 ng/L (ampicillin, chloramphenicol and vancomycin) and 100 ng/L (nitrofurantoin, teicoplanin and tigecycline). Among the analytes, a significant variability in terms of concentrations and numbers of quantifiable samples was observed (see Table 3). Generally, all the analytes were detected infrequently in surface water (SW IN and SW OUT), and quantifiable concentrations usually did not exceed 10 ng/L (with the exception of vancomycin, up to 53 ng/L). Within the entire dataset (surface water and wastewater), teicoplanin was detected in one sample only (SW OUT), but the concentration was below the limit of quantification (LOQ). Similarly, nitrofurantoin was observed rather infrequently. In two samples of raw hospital sewage water, its average concentration was 9.3 µg/L. No study has been found in the literature reporting on nitrofurantoin levels in sewage water. Goessens et al. published a study on antimicrobial residues in pond water in Flanders, Belgium, where nitrofurantoin was below the limit of detection (LOD) in all samples [36].

Chloramphenicol was observed frequently in hospital wastewater and raw municipal wastewater at concentrations of tens to hundreds of ng/L. Rather high concentrations were found in wastewater from the State Veterinary Institute in Olomouc (usually thousands of ng/L). In most samples of surface water, chloramphenicol was below the LOD. In Shenyang, China, Guan et al. determined 297 ng/L in WWTP influent and less than the LOD in river water samples [37], while Li et al. reported concentrations from below the LOD to 635 ng/L [38]. Camacho-Munoz et al. did not detect chloramphenicol in any samples taken from two WWTPs in Western and Northern Europe [39].

Ampicillin was found rarely in municipal WWTP influent (one case, a low concentration) and occasionally in hospital raw wastewater samples with a high average concentration of 48,000 ng/L. Li et al. reported concentrations of ampicillin in Hong Kong municipal WWTP samples from below the LOD to 389 ng/L [40]. On the other hand, Gros et al. did not detect ampicillin in any samples taken from hospital and municipal WWTPs in Girona, Spain (LOD ~5 ng/L) [41]. Kim et al. detected ampicillin in the effluent from a municipal WWTP in South Korea, but the concentration was below the LOQ (2.9 ng/L) [42].

Linezolid was quantified rather often in both hospital (up to thousands of ng/L) and municipal wastewater (usually ones or tens of ng/L). Angeles et al. reported up to 88 ng/L in river water in Dhaka, Bangladesh [43]. Kulkarni et al. studied concentrations of linezolid in WWTPs processing combined domestic and hospital wastewater in the USA and found rather high concentrations ranging from 3 to 61 µg/L in influent and from below the LOD to 22 µg/L in effluent samples [44]. Craddock et al. investigated linezolid occurrence in greywater used for irrigation in the West Bank territory, finding linezolid in approximately half of the samples, with concentrations of up to 13 ng/L in raw greywater and up to 2 ng/L after its treatment [45].

Clindamycin was found and quantified in almost all the samples of wastewater. While the mean concentrations for raw and treated hospital wastewater were 1200–1500 ng/L, only tens of ng/L were detected in municipal raw wastewater. Gros et al. determined the clindamycin concentrations in municipal and hospital wastewater in Girona, Spain and found 21–57 ng/L and 184–1465 ng/L, respectively [41]. Tylova et al. found 27–151 ng/L and less than the LOD to 102 ng/L in influent and effluent samples, respectively, collected in six WWTPs in the Czech Republic [46]. Rossmann et al. monitored the clindamycin occurrence in a WWTP in Dresden, Germany, finding concentrations of 11–163 ng/L and 20–882 ng/L in influent and effluent samples, respectively [47]. In the Miami River, concentrations of 5–25 ng/L were found [48].

Tetracycline and tigecycline were observed only sparingly in both municipal and hospital wastewater. While literature data on the occurrence of tigecycline in the aquatic environment are not available, tetracycline belongs to frequently monitored substances. For example, Serra-Compte et al. did not detect tetracycline in any wastewater or fresh water samples at two sampling locations in Spain [49]. Similarly, Tylova et al. did not detect tetracycline in any WWTP influent or effluent samples at six locations in the Czech Republic [46]. Li et al. determined 271 ng/L in the influent of a Hong Kong municipal WWTP [40]. Dinh et al. investigated the tetracycline concentrations in river water at six locations in the Seine River basin, finding the substance in one sample only at a low concentration of 7.4 ng/L [50].

Vancomycin was present in most samples of both municipal and hospital wastewater. Concentrations in municipal wastewater ranged from tens to low hundreds of ng/L. On the other hand, concentrations in many hospital wastewater samples (especially UHO IN and UHO OUT) were very high, often exceeding 10,000 ng/L. Dinh et al. studied sewage vancomycin concentrations in Fontenay-les-Briis, France, reporting 0.22–10.6 µg/L in hospital wastewater and less than the LOD in domestic wastewater [51]. In the municipal WWTP in Dresden, Germany, Rossmann et al. determined up to 664 ng/L in the influent (quantifiable in 8% of samples) and up to 348 ng/L in the effluent (quantifiable in 2% of samples) [47]. Giebultowicz et al. investigated vancomycin concentrations in two WWTPs in Poland and found mean concentrations of 350 and 3200 ng/L in influent and 114 and 162 ng/L in effluent samples, respectively [52]. In samples of surface water of the Vistula River, Poland, receiving effluent from the Warsaw WWTP, Giebultowicz et al. determined concentrations of 29–117 ng/L [53]. Dinh et al. reported 90 ng/L of vancomycin at one location in the Seine River basin (downstream of a WWTP); at five other sampling points, vancomycin was not detected [50]. Tran et al. studied occurrence of vancomycin in municipal canals and lakes in Hanoi, Vietnam [54]. In the canals, vancomycin was found in most samples at concentrations of up to 249 ng/L, while the lakes contained up to 26 ng/L of the substance. Omotola et al. reported rather high concentrations (10–22 µg/L) in the Msunduzi River, South Africa [55].

Erythromycin was detected and quantified in many samples of municipal and hospital wastewater at concentrations usually of ones to tens of ng/L. Li et al. reported concentrations between 38 and 217 ng/L in WWTP samples in Hong Kong [40]. Tylova et al. determined erythromycin concentrations between 9 and 249 ng/L in the influents and less than the LOD to 204 ng/L in the effluents of six WWTPs in the Czech Republic [46]. Pascale et al. did not detect erythromycin in samples from Potenza WWTP, Italy (LOD = 0.7 ng/L) [56]. Senta et al. investigated erythromycin occurrence in Zagreb, Croatia, reporting concentrations of less than the LOD to 40 ng/L in municipal wastewater and up to 10 µg/L in river water receiving effluent from a pharmaceutical production plant [57].

### 3.2. Determining PNECs for the Tested Antibiotics in Samples

Throughout the study period, the PNEC, defined by Bengtsson-Palme & Larsson [23] as a threshold below which adverse effects of antimicrobials are most unlikely to occur during exposure in the aquatic environment, was exceeded in 32% of samples of all the tested antibiotics, namely five out of the 10 antibiotics, most frequently in vancomycin (29 times—16% of the tested samples) (see Table 3).

For ampicillin, concentrations exceeding the PNEC (250 ng/L) were detected in only four samples of raw wastewater from the Military Hospital Olomouc; that is, 3% of them all. In their study assessing PNECs in 13 European WWTPs, Rodriguez-Mozaz et al. detected ampicillin in only one plant located in Ireland [19].

Concentrations of clindamycin in hospital wastewater often contained hundreds to thousands of ng/L, and the PNEC (1000 ng/L) was exceeded in 22 samples out of 90. Those were raw wastewater samples collected in both hospitals, as well as University Hospital Olomouc WWTP effluent samples. In the study by Rodriguez-Mozaz et al., the PNEC for clindamycin was not exceeded in any of the European WWTPs included [19].

Tetracycline concentrations in the tested samples were below the PNEC (1000 ng/L); this is likely because of the very limited use of this pharmaceutical in human medicine in the Czech Republic. The PNEC was exceeded in only one case, a sample of raw wastewater from the Military Hospital Olomouc. Similarly, low tetracycline concentrations below the PNEC were reported by Rodriguez-Mozaz et al. 2020 [19].

Tigecycline was detected in eight wastewater samples (from a total of 180 samples); in two cases of hospital wastewater, its concentration exceeded the PNEC (1000 ng/L). In our opinion, this is the first description of this antibiotic being present in wastewater, since literature data on the occurrence of tigecycline in the aquatic environment are not available.

Vancomycin was one of the most frequently detected antibiotics in the monitored samples obtained from wastewater and the Morava River. As for raw wastewater samples from the hospitals, the PNEC for this antibiotic (8000 ng/L) was most frequently exceeded in the University Hospital Olomouc samples: both UHO IN and UHO OUT samples. The high detection rates may be explained by the fact that the hospital WWTP takes wastewater from the Department of Hemato-Oncology, where vancomycin is frequently administered to patients with infections caused by multi-resistant staphylococci and strains of *Enterococcus faecium*. Vancomycin is used almost exclusively in the hospital setting [51,54], and that is probably why its excessive concentrations were only seen in the hospital sampling sites.

### 3.3. Molecular Biological Characteristics of VRE in Wastewater and Surface Water

Over the study period, a total of 18 unique VRE strains of *Enterococcus faecium* were obtained from water samples, of which 16 were collected from WWTPs and two from the Morava River. Throughout the study, *E. faecalis* strains were not isolated in wastewater. Additionally, in UHO patients, vancomycin-resistant *E. faecium* strains only were isolated. As seen from Table 4, the highest proportion of samples were from the municipal WWTP effluent, mainly collected between June 2019 and July 2020. By contrast, no surface water samples collected past the effluent of the municipal WWTP or State Veterinary Institute in Olomouc wastewater samples yielded VRE.

Sixteen VRE isolates detected in 144 wastewater samples (11%) represented a proportion higher than that in a Portuguese study showing 17 isolates out of 499 WWTP samples (3%) [58]. Rosenberg Goldstein et al. reported 12 VRE isolates out of 44 WWTP samples (27%) in the USA [59]. A Swedish study comprising 118 wastewater and surface water samples identified 33 VRE isolates, with a 40% prevalence of VRE isolates in WWTP samples [60].

The present study found two VRE isolates in 36 surface water samples (6%). Iversen et al. reported one VRE out of 37 surface water samples (3%) [60].

For all isolated VRE, susceptibility to antibiotics was determined, including minimum inhibitory concentrations (MICs) (Table 5). The results showed 100% resistance to ampicillin, penicillin and erythromycin. All but two strains were resistant to clindamycin and teicoplanin (one isolate each). High susceptibility was detected to tigecycline (90%) and linezolid (100%). In a study by Rosenberg Goldstein et al., VRE detected in USA WWTPs were resistant to erythromycin, penicillin, tetracycline, vancomycin, ciprofloxacin and streptomycin and susceptible to linezolid [59]. A similar range of resistance was reported by Araújo et al. [58].

Using PCR and subsequent gel electrophoresis, the vanA and vanB genes were found in 17 and one VRE isolates, respectively. Additional genetic analyses revealed genes encoding the enterococcal surface protein and hyaluronidase. In a series of 18 isolates, the esp (= 4), hyl (= 6) and esp + hyl (= 3) genes were identified. No virulence factors were found in five VRE. The esp gene was also detected in vanB VRE obtained from WWTPs in England [61].

Table 6 shows the prevalence of selected genes of resistance to tetracyclines and macrolides and their comparison with MICs of the tested antibiotics. In isolates obtained from wastewater samples, the tet(M) + erm(B) (five isolates) and erm(B) genes were most common (five isolates each). The tetK, tet(O), tet(S), tet(X), erm(A) and mef(A/E) genes were not found. Similarly, Araújo et al. found that, in vancomycin-resistant *E. faecium* from WWTPs, the most frequent was the tet(M)+ erm(B) gene combination [58].

In 16 VRE, two different aminoglycoside resistance genes of the following combination were identified: aac(6′)-Ie-aph(2′)-Ia + aph(3′)-IIIa (11 isolates) and aac(6′)-Ie-aph(2′)-Ia (3 isolates) and aph(3′)-IIIa (2 isolates). No aminoglycoside resistance genes were found in two VRE. The presence of the aac6′-aph2′′ was also reported by Araújo et al., namely in two out of 17 VRE from WWTPs that showed a high resistance to gentamicin [58].

The C-terminal region of the pbp5 gene was investigated by PCR and subsequent direct sequencing in all ampicillin-resistant VRE strains. The obtained sequences were compared with the reference sequence included in GenBank (accession no. X84860), and the polymorphism of the specific area was analyzed. Three alleles named A–C, encoding different amino acid changes, were identified (Table 7). Specific amino acid changes in the C-terminal region of the PBP5 at positions 466´ (the insertion), 485, 496, 499, 525 and 629, probably associated with ampicillin resistance, were detected.

### 3.4. Pulsed-Field Gel Electrophoresis of 18 Isolates

**Figure 1:**
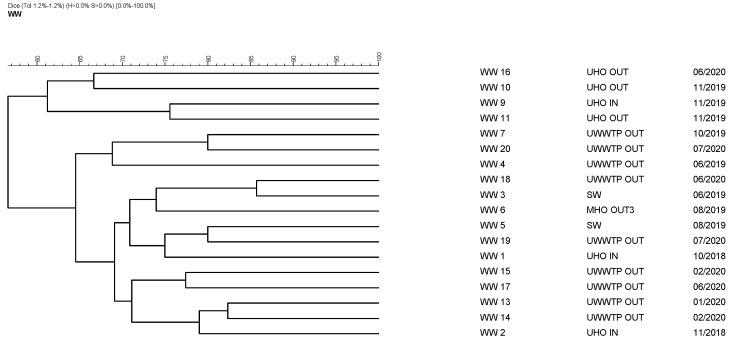
Pulse-field gel electrophoresis dendrogram of 18 VRE *E. faecium* isolates.

The clonal relationship among the isolates was assessed using PFGE. Figure 1 shows that, over the study period, no clonal groups were identified. Similarly, no identical strains are apparent in the same sampling sites. Thus, all tested isolates showed unique restriction profiles. By contrast, Sahlström et al. did find identical strains among VRE from a WWTP [62]. This may illustrate the ability of enterococci to persist in a WWTP setting.

### 3.5. Effect of Vancomycin on VRE Prevalence

The resulting vancomycin concentrations in wastewater and Morava River water samples obtained from individual sampling sites over the study period varied. The highest concentrations were found in both influent and effluent University Hospital Olomouc wastewater samples, including the most common cases of PNEC being exceeded during the entire study period. In Military Hospital Olomouc sewer drain samples, only very low vancomycin concentrations, or concentrations below the PNEC, were recorded; the only two exceptions were February and November 2019. In samples collected from the other sampling sites (i.e., Morava River before the influent and past the effluent of the Municipal WWTP, Olomouc Municipal WWTP influent/effluent and State Veterinary Institute in Olomouc WWTP effluent), only low concentrations not exceeding the PNEC were detected throughout the entire study.

**Figure 2:**
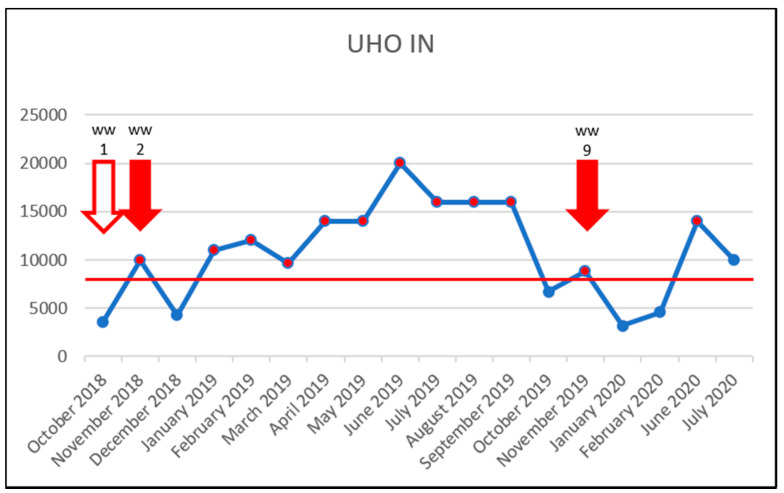
Detection of VRE in the UHO IN samples during the study. x-axis: vancomycin concentration in ng/L/y-axis: collection dates; arrow outline—VRE detection and sample identification; solid red arrow—VRE detection and VRE concentration exceeding the PNEC and sample identification; red horizontal line—the PNEC for vancomycin (8000 ng/L).

**Figure 3:**
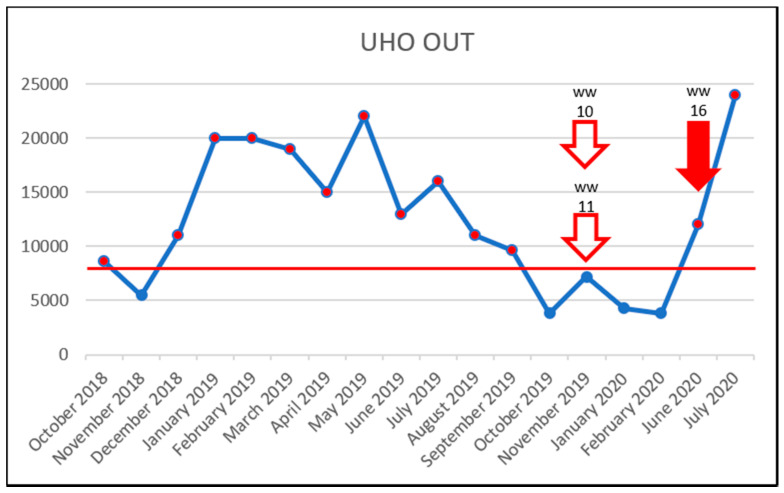
Detection of VRE in the UHO OUT samples during the study. x-axis: vancomycin concentration in ng/L/y-axis: collection dates; arrow outline—VRE detection and sample identification; solid red arrow—VRE detection and VRE concentration exceeding the PNEC and sample identification; red horizontal line—the PNEC for vancomycin (8000 ng/L).

To investigate the effect of increased vancomycin concentrations on the prevalence of VRE, the results were plotted in time charts below but only for sampling sites in which VRE were detected. The red line shows the PNEC; below which, the antibiotic probably has no adverse effects in the ecosystem [19]. For vancomycin, this was set at 8000 ng/L, in accordance with the study by Bengtsson-Palme & Larsson [23]. The red arrow outline shows the month in which VRE were detected. The solid red arrow means a concentration above the PNEC and detection of VRE. Thus, a correlation between increased vancomycin concentration (above the PNEC) and VRE detection was not confirmed. Even though, in the University Hospital Olomouc samples, the PNEC was exceeded almost throughout the entire study, only three VRE were detected in both the influent and effluent WWTP samples (Figure 2 and Figure 3). In the case of samples WW2, WW9 and WW16, there is a theoretical possibility of increased vancomycin concentrations being correlated with detected VRE. However, those were only three out of 18 VRE identified during the entire study period. The source of VRE in the University Hospital Olomouc wastewater samples is likely to be patients’ biological material—in particular, that from the department of Hemato-Oncology. In the hospital, only wastewater from the Oncology and Hemato-Oncology Departments was brought to the WWTP. In their 2020 study, Hricová et al. reported that 7% of hemato-oncological patients carried VRE in their gastrointestinal tract, and in all cases, the species identified was *E. faecium* [8]. The results of VRE susceptibility testing showed a very high susceptibility to linezolid (100%) and tigecycline (90%) and, conversely, 100% resistance to ampicillin and 95% in the case of teicoplanin. These results were completely consistent with the resistance of VRE isolated in hemato-oncological patients [8].

**Figure 4:**
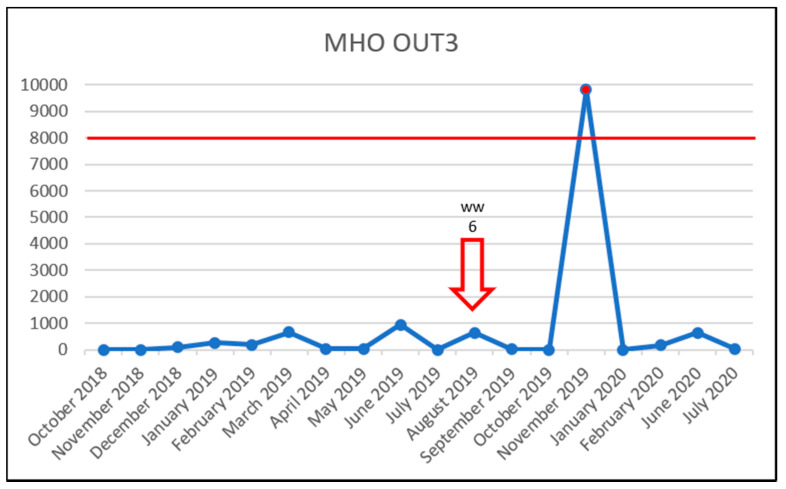
Detection of VRE in the MHO OUT3 samples during the study. x-axis: vancomycin concentration in ng/L/y-axis: collection dates; arrow outline—VRE detection and sample identification; solid red arrow—VRE detection and VRE concentration exceeding the PNEC and sample identification; red horizontal line—the PNEC for vancomycin (8000 ng/L).

In the Military Hospital Olomouc wastewater samples, there was only one case of the vancomycin concentration exceeding the PNEC, and VRE was detected on only one occasion (Figure 4).

**Figure 5:**
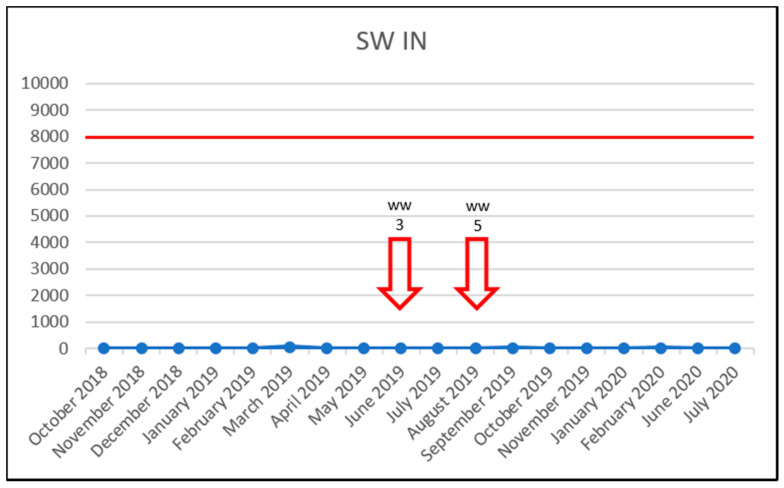
Detection of VRE in the surface water (SW IN) samples during the study. x-axis: vancomycin concentration in ng/L/y-axis: collection dates; arrow outline—VRE detection and sample identification; solid red arrow—VRE detection and VRE concentration exceeding the PNEC and sample identification; red horizontal line—the PNEC for vancomycin (8000 ng/L).

In the surface water, the PNEC was never exceeded during the study period, and two VRE isolates were identified (Figure 5).

**Figure 6:**
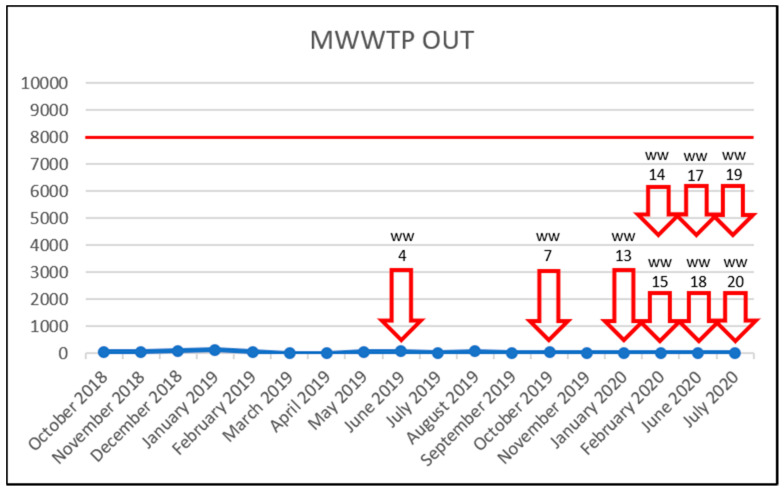
Detection of VRE in the MWWTP OUT samples during the study. x-axis: vancomycin concentration in ng/L/y-axis: collection dates; arrow outline—VRE detection and sample identification; solid red arrow—VRE detection and VRE concentration exceeding the PNEC and sample identification; red horizontal line—the PNEC for vancomycin (8000 ng/L).

Most frequently, VRE were detected in the municipal WWTP effluent samples. A total of nine unique VRE were identified; however, the PNEC set at 8000 ng/L was never exceeded throughout the study (Figure 6). Once again, the vancomycin concentration and VRE detection were not correlated in this sampling site. The frequent VRE detection in the WWTP effluent samples may be theoretically explained by the ability of (not only) enterococci to persist in the WWTP environment, contributed to by favorable conditions such as adequate temperature, pH and nutrition [13,16,17,62].

## 4. Conclusions

The study showed the presence of VRE in wastewater. All isolates were *Enterococcus faecium*, and all but one were of the VanA phenotype. At the same time, 16% (32/180) of the wastewater and Morava River samples were shown to contain vancomycin at concentrations above the PNEC, namely 8000 ng/L. Most frequently, the PNEC was exceeded in both the influent and effluent University Hospital Olomouc WWTP samples (29/32). However, detection of VRE in the wastewater samples was not correlated with the vancomycin concentrations. VRE detected in the wastewater and vancomycin concentration above the PNEC were found in only three out of 18 cases, exclusively samples collected from the University Hospital Olomouc. Thus, no direct relationship between vancomycin residues and the presence of VRE was confirmed.

## Figures and Tables

**Table 1:** Description of the sampling sites and identification of wastewater and surface water samples.

Sample Identification	Acronym Explanation	Sample Type
MHO OUT1	Military Hospital Olomouc—hospital sewer drain 1	Raw sewage
MHO OUT2	Military Hospital Olomouc—hospital sewer drain 2	Raw sewage
MHO OUT3	Military Hospital Olomouc—hospital sewer drain 3	Raw sewage
UHO IN	University Hospital Olomouc—hospital wastewater treatment plant influent	Raw sewage
UHO OUT	University Hospital Olomouc—hospital wastewater treatment plant effluent	Treated sewage
MWWTP IN	Olomouc Municipal Wastewater Treatment Plant influent	Raw sewage
MWWTP OUT	Olomouc Municipal Wastewater Treatment Plant effluent	Treated sewage
SVI OUT	State Veterinary Institute wastewater treatment plant effluent	Treated sewage
SW IN	Surface water (Morava River)—before the Municipal Wastewater Treatment Plant influent	Surface water
SW OUT	Surface water (Morava River)—past the Municipal Wastewater Treatment Plant effluent	Surface water

**Table 2:** Nucleotide sequences of the primer sets used to amplify the *pbp5* gene.

Gene	Primer Name	Primer Sequence (5′ to 3′)	Amplicon Size (bp)
*pbp5*	PBP5 6F	AACCGGTGATCTTCTTGCG	756
	PBP5 6R	TTGATTTCCGCTGTACCAGT	
	PBP5 11F	GGGCTTAAATGGCAAAAAGA	694
	PBP5 11R	ATTGATAATTTTGGTTGAGGTAT	

**Table 3:** Mean concentrations of antimicrobial residues detected in surface water and wastewater.

Antibiotic	PNEC ng/L	Mean Concentration ^a,b^ (ng/L)					No. of Samples Exceeding PNEC ^c^					
		Raw Waste-Water (Hospital) ^d^	Raw Waste-Water (Municipal)	Treated Waste-Water (Hospital)	Treated Wastewater (Municipal)	Surface Water	Raw Waste-Water (Hospital)	Raw Waste-Water (Municipal)	Treated Waste-Water (Hospital)	Treated Waste-Water (Municipal)	Surface Water	Total
nitrofurantoin	64,000	9300 (2)	**-**	**-**	**-**	**-**	**-**	**-**	**-**	**-**	**-**	**-**
chloramphenicol	8000	890 (69)	160 (18)	240 (8)	10 (4)	16 (4)	**-**	**-**	**-**	**-**	**-**	**-**
linezolid	8000	1200 (22)	48 (9)	980 (16)	10 (10)	3 (5)	**-**	**-**	**-**	**-**	**-**	**-**
ampicillin	250	48,000 (5)	92 (1)	38 (1)	**-**	9 (1)	4	**-**	**-**	**-**	**-**	4
clindamycin	1000	1500 (66)	81 (16)	1200 (17)	38 (18)	2 (9)	16	**-**	6	**-**	**-**	22
tetracycline	1000	2700 (5)	37 (2)	120 (4)	8 (1)	7 (4)	1	**-**	**-**	**-**	**-**	1
tigecycline	1000	800 (6)	280 (1)	**-**	950 (1)	**-**	2	**-**	**-**	**-**	**-**	2
vancomycin	8000	4700 (58)	140 (17)	13,000 (18)	45 (16)	25 (6)	16	**-**	13	**-**	**-**	29
erythromycin	1000	67 (35)	36 (18)	52 (10)	12 (13)	**-**	**-**	**-**	**-**	**-**	**-**	**-**

^a^ In parentheses, the number of samples in which the analyte could be quantified (concentration > LOQ) is indicated; the mean concentrations were calculated using the number of samples with quantifiable concentrations. Total no. of samples: raw wastewater (hospital) = 90, raw wastewater (municipal) = 18, treated wastewater (hospital) = 18, treated wastewater (municipal) = 18 and surface water = 36. ^b^ “**-**” indicates that no numerical concentration value was available. ^c^ “**-**” indicates that no sample exceeded the PNEC. ^d^ Includes raw wastewater from the State Veterinary Institute in Olomouc.

**Table 4:** All VRE obtained from the sampling sites.

Laboratory Sample Identification	Sampling Site	Sample Type	Collection Date
WW1	UHO IN	raw sewage	10/2018
WW2	UHO IN	raw sewage	11/2018
WW3	SW IN	surface water	06/2019
WW4	UWWTP OUT	treated sewage	06/2019
WW5	SW IN	surface water	08/2019
WW6	MHO OUT3	raw sewage	08/2019
WW7	UWWTP OUT	treated sewage	10/2019
WW9	UHO IN	raw sewage	11/2019
WW10	UHO OUT	treated sewage	11/2019
WW11	UHO OUT	treated sewage	11/2019
WW13	UWWTP OUT	treated sewage	01/2020
WW14	UWWTP OUT	treated sewage	02/2020
WW15	UWWTP OUT	treated sewage	02/2020
WW16	UHO OUT	treated sewage	06/2020
WW17	UWWTP OUT	treated sewage	06/2020
WW18	UWWTP OUT	treated sewage	06/2020
WW19	UWWTP OUT	treated sewage	07/2020
WW20	UWWTP OUT	treated sewage	07/2020

**Table 5:** Minimum inhibitory concentrations of the tested antibiotics in a series of 18 VRE.

Laboratory Sample Identification	PEN	LNZ	AMP	TIG	CMP	TET	ERY	CLI	VAN	TEI	FUR
WW1	4	1.5	16	0.03	2	0.25	16	8	8	2	64
WW2	4	2	16	0.03	2	16	16	8	32	32	512
WW3	0.5	0.25	16	0.03	2	16	8	4	64	64	32
WW4	2	1.5	16	0.03	1	16	8	4	64	32	32
WW5	4	0.5	16	0.03	2	0.25	16	8	64	8	64
WW6	4	0.25	16	0.03	2	16	16	8	64	8	64
WW7	8	1	16	4	1	16	8	4	8	4	32
WW9	2	1.5	16	0.5	2	0.5	4	4	32	2	64
WW10	2	1.5	8	0.03	2	16	4	4	32	8	32
WW11	2	1.5	16	0.03	2	0.5	4	4	8	0.5	32
WW13	2	1	16	0.03	2	16	8	16	16	16	8
WW14	2	1.5	16	0.03	2	16	8	2	16	16	16
WW15	2	1.5	16	0.03	4	16	8	16	16	16	16
WW16	2	2	16	0.03	4	16	8	4	16	32	256
WW17	2	1	16	0.06	1	16	4	0.06	32	16	32
WW18	2	0.03	16	0.03	4	0.5	8	4	16	8	256
WW19	2	0.03	16	0.03	2	8	4	8	16	8	32
WW20	2	0.03	16	0.03	4	0.25	4	8	64	8	64

Legend: values shown in mg/L; PEN—penicillin, LNZ—linezolid, AMP—ampicillin, TIG—tigecycline, CMP—chloramphenicol, TET—tetracycline, ERY—erythromycin, CLI—clindamycin, VAN—vancomycin, TEI—teicoplanin and FUR—nitrofurantoin.

**Table 6:** Distribution of tetracycline and macrolide genes among the VRE isolates.

No. of Isolates	Presence of Gene(s)									MIC Range (µg/mL)		
	tet (K)	tet (L)	tet (M)	tet (O)	tet (S)	tet (X)	erm (A)	erm (B)	mef (A/E)	ERY	CLI	TET
3	-	+	+	-	-	-	-	+	-	4–8	4–16	16
4	-	+	+	-	-	-	-	-	-	4–16	0.06–8	0.25–16
5	-	-	+	-	-	-	-	+	-	4–16	4–16	0.25–16
1	-	-	+	-	-	-	-	-	-	8	4	16
5	-	-	-	-	-	-	-	+	-	4–16	4–8	0.25–16

Legend: ERY—erythromycin, CLI—clindamycin and TET—tetracycline.

**Table 7:** pbp5 allele polymorphisms in the C-terminal region of the VRE isolates.

*pbp5* Allele		Amino Acid Changes at Position																	MIC Range of Ampicillin (µg/mL)
	No. of Isolates	461	**466**	470	471	**485**	487	**496**	497	**499**	**525**	581	**586**	595	622	**629**	631	634	
X84860		Q	-	H	V	M	Q	N	F	A	E	I	V	E	E	E	G	N	-
A	11	K	S	Q	V	A	Q	K	F	T	D	I	V	E	E	V	G	N	16
B	6	Q	S	Q	V	A	Q	K	F	T	D	I	V	E	E	V	G	N	16
C	1	Q	D	Q	V	A	Q	K	F	I	D	I	V	E	E	V	G	N	8

Bold letters indicate amino acid positions potentially significant in beta-lactam resistance.

## Data Availability

All data presented in this study are included in this article.

## Supplementary Materials

The following are available online at https://www.mdpi.com/article/10.3390/life11121403/s1: Figure S1: Sampling sites in Olomouc City, Figure S2. A chromatogram of a standard mixture of antibiotics, positive ionization mode, Figure S3: A chromatogram of a standard mixture of antibiotics, negative ionization mode, Table S1: Limit of detection (LOD) and lower limit of quantitation (LLOQ) for the antibiotic residues, Table S2: Supplementary information. The list of retention times (tR) and SRM transitions, Text S1, Text S2.
