# Supplementary material for: Prevalence of Vancomycin-Resistant Enterococci and Antimicrobial Residues in Wastewater and Surface Water
Source: Life (Basel). 2021 Dec 15;11(12):1403. doi: 10.3390/life11121403 (PMC8706104; doi:10.3390/life11121403)
Supplement: Supplementary file 1 [file life-11-01403-s001.zip › life-1475857-supplementary.pdf]

life

Supplementary Materials

Prevalence of Vancomycin-Resistant Enterococci and Antimicrobial Residues in Wastewater and Surface Water

Kristýna Hricová [1,*], Magdaléna Röderová [1], Petr Fryčák [2], Volodymyr Pauk [2], Ondřej Kurka [2], Kristýna Mezerová [1], Taťána Štosová [1], Jan Bardoň [1], David Milde [2], Pavla Kučová [1] and Milan Kolář [1]

S1 text

The UHO-WWTP uses a combination of mechanical and biological treatment processes with subsequent water disinfection using chlorine dioxide. Treated hospital wastewater effluent goes directly into the urban MWWTP system.

The MWWTP uses a combination of mechanical and biological processes and treats domestic, industrial and health care wastewater of the town and the surrounding areas. The biological stage is designed as the so-called R-D-N system (regeneration-denitrification-nitrification) with post-denitrification and post-aeration systems; the effluent goes directly into the river. The positions and distances of sampling sites are shown in the figure 1 (Google Maps / 01.12.2021 / 10:00) :

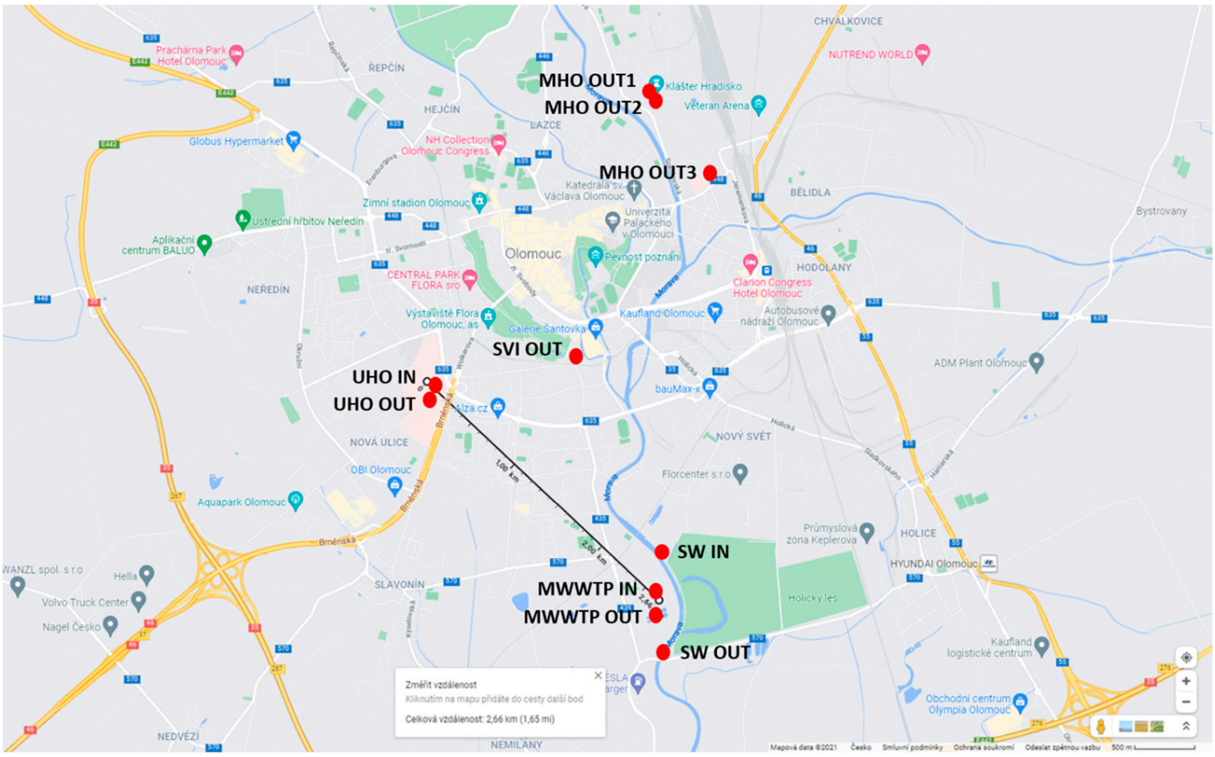

Figure S1. Sampling sites in Olomouc City.

Table S1. Limit of detection (LOD) and lower limit of quantitation (LLOQ) for the antibiotic residues. The limits were derived from measurements of calibration samples. LOD represents the lowest calibration sample concentration where (i) a discernible chromatographic peak of the analyte could be observed and (ii) the peak area was at least twice higher then area of a peak observed in the blank sample or internal standard sample (if such peak was present). LLOQ represents the lowest calibration sample concentration that (i) lied on the linear portion of the calibration curve (R2 > 0.99) and (ii) RSD was less than 15% and (iii) bias was less than 15% from the respective true concentration value of the calibration sample.

Antibiotic	LOD ng/L	LLOQ ng/L
nitrofurantoin	10	20
chloramphenicol	5	5
linezolid	0.5	0.5
ampicillin	5	5
clindamycin	0.5	0.5
tetracycline	0.5	2.5
tigecycline	20	50
vancomycin	5	5
erythromycin	0.5	0.5

Table S2. Supplementary information. The list of retention times (t_R) and SRM transitions.

Substance	t_R, min	Precursor Ion	Quantifier Ion (collision energy, V)	Qualifier Ion (collision energy, V)
Nitrofurantoin	2.65	237.0	151.9 (15)	123.9 (20)
Nitrofurantoin $^{13}C_3$	2.65	240.0	151.9 (15)	-
Chloramphenicol	3.24	320.9	152.2 (15)	257.1 (10)
Chloramphenicol d_5	3.23	325.9	262.1 (10)	-
Teicoplanin	3.32	939.0	678.2 (21)	781.4 (15)
Linezolid	3.21	338.1	296.1 (18)	195.1 (23/12 *)
Linezolid d_3	3.21	341.1	195.1 (23)	-
Ampicillin	2.70	350.0	192.0 (20/10 *)	105.9 (25)
Ampicillin d_5	2.69	355.0	110.9 (25)	-
Clindamycin	3.20	425.1	126.2 (26)	377.1 (16/10 *)
Clindamycin d_3	3.20	428.1	129.2 (26)	-
Tetracycline	2.53	445.1	154.0 (35/25 *)	410.3 (22)
Tetracycline d_6	2.53	451.1	416.3 (22)	-
Tigecycline	1.90	586.3	569.2 (25)	513.2 (20)
Tigecycline d_9	1.90	595.3	578.2 (25)	-
Vancomycin	2.07	725.2	100.2 (35/12 *)	144.1 (15)
Anhydro-Erythromycin	3.53	716.0	557.9 (15)	157.9 (25)
Anhydro-Erythromycin ^{13}C, d_3	3.53	720.0	162.1 (25)	-

* Specific transitions with decreased sensitivity for measurement of high concentrations.

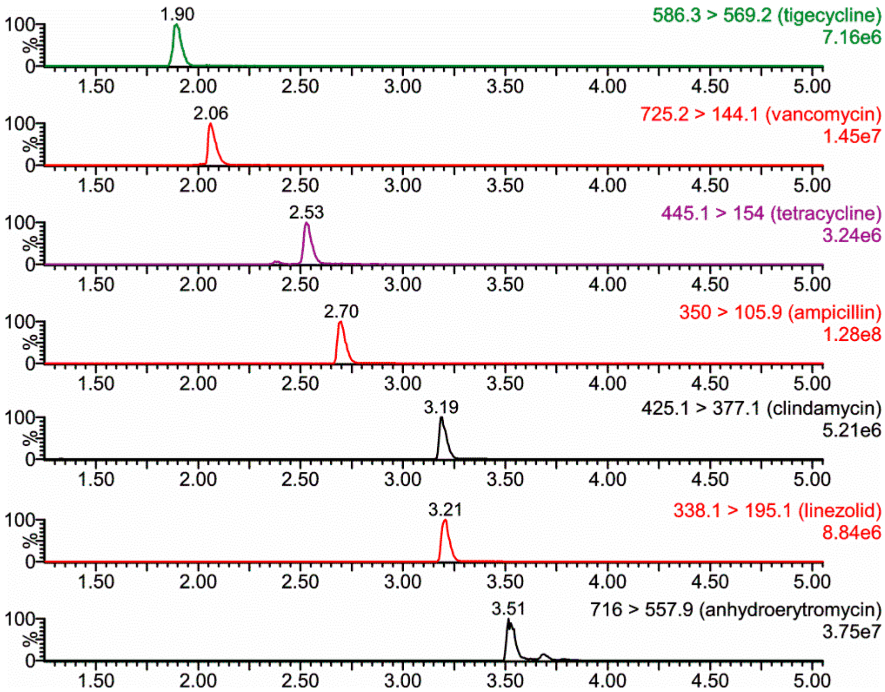

Figure S2. A chromatogram of a standard mixture of antibiotics, positive ionization mode.

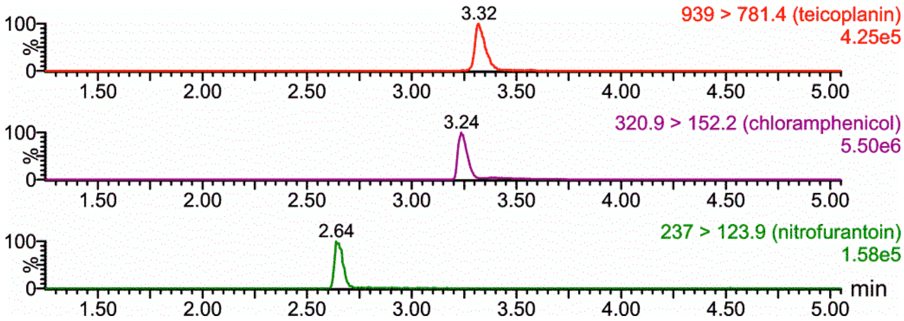

Figure S3. A chromatogram of a standard mixture of antibiotics, negative ionization mode.

S2 text

The reaction mixture contained complete reaction buffer with $MgCl_2$, containing 100 mmol/L Tris-HCl (pH 8.8), 500 mmol/L KCl, 1% Triton X-100, 15 mmol/L $MgCl_2$ (Top-Bio, Czech Republic), 1 U of Taq DNA polymerase (Top-Bio), 0.8 µmol/L primer concentration for each primer, 80 µmol/L concentration of deoxynucleoside triphosphates and 1 µL of template DNA. The PCR was run under the following conditions: initial denaturation at 94 °C for 5 min, 35 cycles of denaturation (94 °C for 30 s), annealing (57 °C for PBP5 6F6R primers / 54 °C for PBP5 11F11R for 30 s), extension (72°C for 60 s), and final extension at 72°C for 7 min. PCR products were purified using the QIAquick purification kit (QIAGEN, Germany) under condition recommended by the manufacturer.
